# First-Principles Insights into Highly Sensitive and Reusable MoS_2_ Monolayers for Heavy Metal Detection

**DOI:** 10.3390/mi15080978

**Published:** 2024-07-30

**Authors:** Jiayin Wu, Zongbao Li, Tongle Liang, Qiuyan Mo, Jingting Wei, Bin Li, Xiaobo Xing

**Affiliations:** 1 Department of Engineering Technology, Guangdong Open University, Guangzhou 510091, China; wujiayin@m.scnu.edu.cn (J.W.); bli@gdrtvu.edu.cn (B.L.); jtwei@gdrtvu.edu.cn (J.W.); 2 Centre for Optical and Electromagnetic Research, South China Academy of Advanced Optoelectronics, South China Normal University, Guangzhou 510006, China; 3 Ministry of Education Key Laboratory of Textile Fiber Products, School of Materials Science and Engineering, Wuhan Textile University, Wuhan 430220, China; 4 School of Materials and Chemistry Engineering, Tongren University, Tongren 554300, China; 5 School of Artificial Intelligence, Guangdong Vocational College of Post and Telecom, Guangzhou 510630, China; liangtongle@gupt.edu.cn; 6 Big Data Engineering College, Kaili University, Kaili 556011, China; 2014020983@kluniv.edu.cn

**Keywords:** MoS_2_ monolayer, heavy metal, DFT, NEGF, sensitivity

## Abstract

This study explores the potential of MoS_2_ monolayers as heavy metal sensors for As, Cd, Hg, and Pb using density functional theory (DFT) and Non-Equilibrium Green’s Function (NEGF) simulations. Our findings reveal that As and Pb adsorption significantly alters the surface structure and electronic properties of MoS_2_, introducing impurity levels and reducing the band gap. Conversely, Cd and Hg exhibit weaker interactions with the MoS_2_ surface. The MoS_2_ monolayer sensors demonstrate exceptional sensitivity for all four target heavy metals, with values reaching 126,452.28% for As, 1862.67% for Cd, 427.71% for Hg, and 83,438.90% for Pb. Additionally, the sensors demonstrate selectivity for As and Pb through distinct response peaks at specific bias voltages. As and Pb adsorption also induces magnetism in the MoS_2_ system, potentially enabling magnetic sensing applications. The MoS_2_ monolayer’s moderate adsorption energy facilitates rapid sensor recovery at room temperature for As, Hg, and Cd. Notably, Pb recovery time can be significantly reduced at elevated temperatures, highlighting the reusability of the sensor. These results underscore the potential of MoS_2_ monolayers as highly sensitive, selective, and regenerable sensors for real-time heavy metal detection.

## 1. Introduction

Heavy metals such as arsenic (As), cadmium (Cd), mercury (Hg), and lead (Pb) are known for their acute toxicity and carcinogenic properties, even at minute concentrations. Furthermore, they resist biodegradation in the environment. Found in water bodies, air, and soil, these pervasive environmental contaminants enter the human body through various pathways, including inhalation, the food chain, and absorption through the skin and gastrointestinal tract. Upon entry, heavy metals react with organic molecules in the body to form metal complexes, altering the original biochemical functions of substances and thus posing significant health risks.

In the environment, heavy metals exist in various forms, including ions, particle-bound (HMp), and elemental (HM0). Traditional detection methods have primarily focused on the ionic and HMp states. However, the elemental form, HM0, presents a challenge due to its low solubility in water or electrolyte solutions, rendering conventional detection methods ineffective [1]. For instance, elemental mercury (Hg0), emitted from coal-fired boiler flue gases, and gaseous cadmium (Cd0), from solid waste incineration, are difficult to measure and capture due to their low solubility and surface affinity, respectively [2,3]. Similarly, elemental lead (Pb0), released into the fuel flue gases with low solubility in water, poses a challenge for purify using wet flue gas desulfurization systems [4]. Elemental arsenic (As0) is also one of the predominant forms in which arsenic exists [5]. Therefore, there is an urgent need to develop sensors capable of real-time detection of HM0.

Recent studies have highlighted the potential of two-dimensional materials in heavy metal adsorption and sensing. M. Ghashghaee et al. discovered that black phosphorene monolayers could adsorb Cd, Pb, Hg, and As, effectively removing these heavy metals from the environment [6]. M. A. Hamed et al. reported that graphene-structured ZnO exhibited adsorption capabilities for Cd [7]. Carbon-based two-dimensional materials have also been employed for heavy metal adsorption. C. Zhao et al. demonstrated that pyridinic nitrogen-doped graphene modified with Pd clusters could effectively adsorb Hg and As [8]. I. Shtepliuk et al. utilized the selectivity of graphene/SiC Schottky diodes for highly sensitive detection of lead atoms [9]. J. Liao et al. observed that single vacancy defects significantly enhanced the adsorption of Cd on graphene [10]. Research by M. Srivastava et al. revealed that boron-modified graphene and copper-modified nitrogen-doped graphene could detect As in water [11]. Furthermore, J. Zou et al. modified glassy carbon electrodes with sulfur-doped graphitic carbon nitride nanosheets, which proved to be sensitive and selective for Pb detection [12].

Among two-dimensional chalcogenides, molybdenum disulfide (MoS_2_) monolayers have attracted attention for their high carrier mobility, tunable bandgap, and extensive specific surface area, positioning them as promising candidates for gas sensing applications [13,14,15,16,17,18,19,20,21]. X. Mu and Zhao et al. investigated the adsorption performance of 1T-MoS_2_ and 2H-MoS_2_ for elemental mercury [22,23]. However, reports on the sensing capabilities of MoS_2_ monolayers for heavy metals are still forthcoming.

To address this knowledge gap, our study employs density functional theory (DFT) to explore the heavy metal sensing capabilities of MoS_2_-based monolayer sensors. The adsorption energies, charge transfer, density of states (DOS), band structure, and Bader analysis of As, Cd, Hg, and Pb on MoS_2_ monolayers have been investigated. The desorption process is predicted to be governed by the recovery time. This paper also measures the current voltage characteristics and sensitivity of the MoS_2_ monolayer when detecting heavy metals. Our findings could pave the way for the development of novel nanosensors for environmental monitoring and facilitate the rapid, selective detection of heavy metal contaminants.

## 2. Materials and Methods

Calculations were conducted using the Vienna Ab initio Simulation Package (VASP) 5.4, which implements spin-polarized density functional theory (DFT) [24,25]. The Perdew–Burke–Ernzerhof (PBE) generalized gradient approximation (GGA) was employed for the exchange-correlation functional [26]. To account for van der Waals interactions within the MoS_2_ monolayer, the DFT-D3 method was utilized [27]. Geometry optimization continued until the forces on all atoms converged below a threshold of 0.02 eV/Å. The total energy convergence criterion was set to 10^−5^ eV. A plane wave energy cutoff of 400 eV was used, and a vacuum layer of 20 Å was applied perpendicular to the 2H-MoS_2_ monolayer to minimize interlayer interactions. Brillouin zone integration was performed using a 4 × 4 × 1 k-point Monkhorst–Pack mesh.

The adsorption energy E_ad_ was computed using the relation:

(1) $$\begin{array}{c} E_{ad}=E_{total}-E_{{MoS}_{2}}-E_{metal} \end{array}$$

where E_total_ is the total energy of the fully relaxed adsorbed system consisting of the MoS_2_ monolayer and the adsorbed metal atom, $E_{{MoS}_{2}}$ is the energy of the pristine MoS_2_ monolayer, and E_metal_ is the energy of the isolated heavy metal atom.

Additionally, quantum transport calculations were conducted using the TRANSIESTA code within the SIESTA 4.1.5 framework, utilizing the non-equilibrium Green’s function method [28]. The Landauer–Büttiker formula was applied to calculate the current-voltage (I-V) characteristics of the MoS_2_ monolayer both before and following heavy metal adsorption under [29]:

(2) $$\begin{array}{c} I\left(V_{b}\right)=\frac{2e}{h}\int_{\mu_{R}}^{\mu_{L}}T\left(E,V_{b}\right)\left[f\left(E-\mu_{L}\right)-f\left(E-\mu_{R}\right)\right] \end{array}$$

where *e* and *h* are the electron charge and Planck’s constant, respectively. I is the current through the central scattering region. *μ* and $f\left(E-\mu \right)$ are the electrochemical potentials and the Fermi–Dirac distribution function, where the subscript *L*/*R* is employed to differentiate between the left and right electrodes, respectively. By employing Green’s function formalism, the transmission coefficient $T\left(E,V_{b}\right)$ at energy *E* and bias voltage *V_b_* was calculated.

For the visualization of crystal structures and differential charge density, the software VESTA 3 was employed [30]. Data post-processing was performed using the tool vaspkit 1.3.3 [31].

## 3. Results and Discussion

### 3.1. Structures

**Figure 1:**
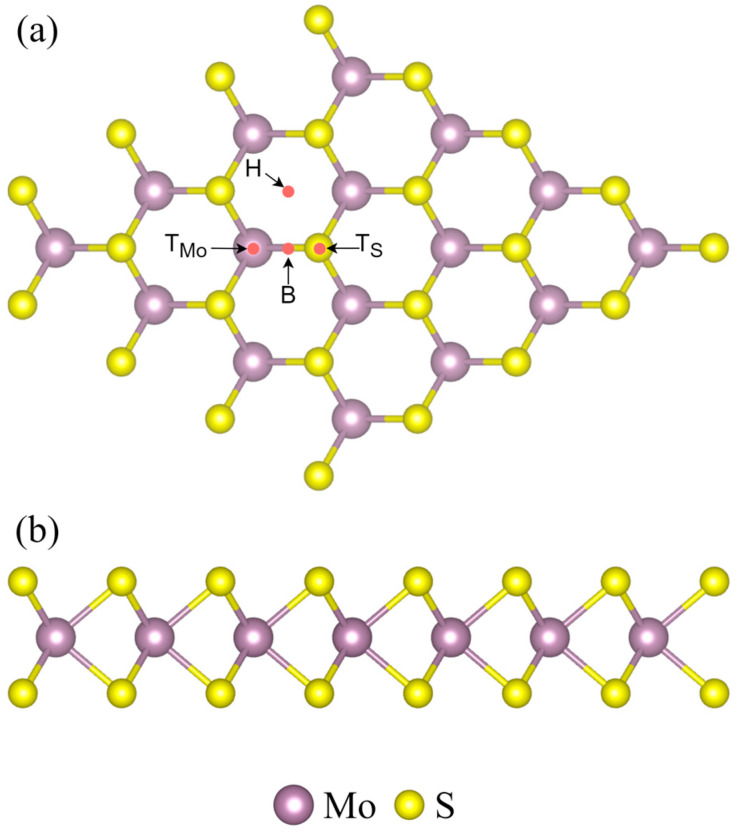
(**a**) The top side view of MoS_2_ monolayer with the position sites (H, B, T_S_, and T_Mo_); (**b**) the side view of MoS_2_ monolayer.

As depicted in Figure 1, the MoS_2_ monolayer supercell exhibits a distinct structural arrangement, where each Mo atom is coordinated with six S atoms, and each S atom is coordinated with three Mo atoms. The Mo–S bond length is measured at 2.417 Å, aligning remarkably with previous studies, which reported bond lengths of 2.424 Å [19]. The bond angles within the MoS_2_ lattice, specifically the S-Mo-S and Mo-S-Mo angles, are 82.60°, forming a hexagonal configuration.

In this study, the energies of heavy metal atoms at four distinct sites on the MoS_2_ surface have been computed: the hexagonal center (H site), atop the Mo atom (T_Mo_ site), atop the S atom (T_S_ site), and the bridge site of the Mo-S bond (B site). These calculations aim to ascertain the most energetically favorable adsorption sites for heavy metals on the MoS_2_ monolayer surface.

**Figure 2:**
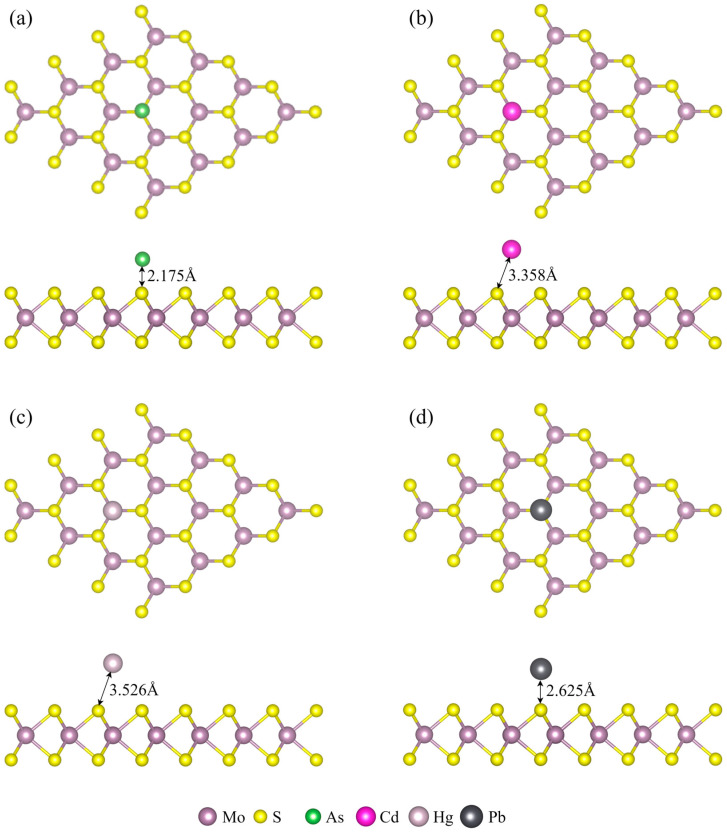
The most stable configuration and Bader charge analysis for (**a**) As, (**b**) Cd, (**c**) Hg, and (**d**) Pb on the MoS_2_ monolayer. Negative numbers indicate loss of electrons, positive numbers indicate gain of electrons.

Figure 2 presents the most stable adsorption configurations of heavy metals—As, Pb, Cd, and Hg—on the MoS_2_ monolayer. Geometric optimization results indicate a predilection for As and Pb to adsorb at the T_S_ site, where they effectively engage with the MoS_2_ surface, achieving the lowest adsorption energies. In contrast, Cd and Hg show a preference for adsorbing on the T_Mo_ site.

During the adsorption process, the heavy metals cause alterations in the underlying bond angles of the MoS_2_ structure. Specifically, following the adsorption of Hg and Cd, the S-Mo-S bond angles increase to 83.15° and 83.36°, respectively. Adsorption of As and Pb results in an increase in the Mo-S-Mo bond angles to 83.52° and 83.74°, respectively. These changes in bond angles indicate the extent of structural perturbation imposed by different heavy metals on the MoS_2_ surface, with Pb exerting the most significant impact, followed by As, Cd, and Hg, in that order. The adsorption distance data show that As and Pb are closer to the MoS_2_ surface, at 2.175 Å and 2.625 Å, respectively, compared to the greater distances for Cd (3.358 Å) and Hg (3.526 Å). This suggests a tighter interaction and stronger interfacial forces between As and Pb with the MoS_2_ surface, leading to more pronounced structural distortions. Conversely, the longer adsorption distances for Cd and Hg suggest relatively weaker interactions and a diminished interfacial coupling effect.

A detailed analysis of the adsorption characteristics of different heavy metal ions on the MoS_2_ monolayer surface has been performed, and the key parameters, including adsorption energy, adsorption distance, Bader charge transfer, and the corresponding changes in the material’s bandgap, are summarized in Table 1.

Among the listed data, the heavy metal elements Cd and Hg exhibit longer adsorption distances, lower corresponding adsorption energies, and smaller charge transfers. This indicates relatively weaker interactions between Cd and Hg with the MoS_2_ monolayer, which are not conducive to forming stable chemisorption states. Conversely, As and Pb demonstrate significantly enhanced adsorption characteristics on the MoS_2_ monolayer. Not only is their adsorption energy significantly reduced, indicating more stable adsorption, but their adsorption distances are also substantially shorter. This implies that the interactions between As and Pb with the MoS_2_ monolayer are more intense, leading to more efficient surface adsorption reactions.

### 3.2. Eletronic Properties

In the exploration of monolayer MoS_2_ as a sensing material, the analysis of its band structure is crucial, particularly the characteristics of the bands near the Fermi level (from −2 eV to 2 eV) which directly influence the material’s conductivity changes and sensing response sensitivity. The theoretical band gap of pristine monolayer MoS_2_ has been calculated to be 1.585 eV.

**Figure 3:**
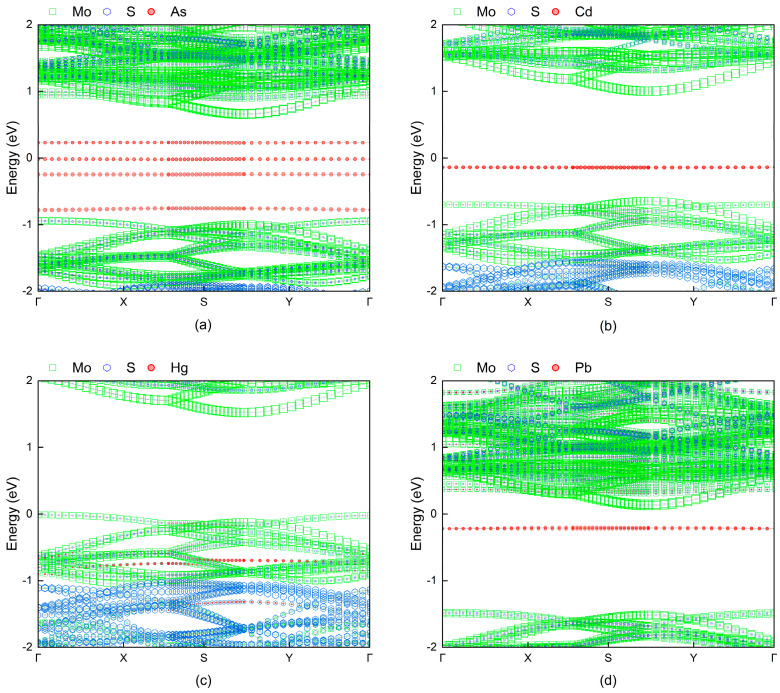
Band structures of (**a**) As, (**b**) Cd, (**c**) Hg, and (**d**) Pb adsorbed onto the MoS_2_ monolayer.

Upon adsorption of As atoms on the MoS_2_ monolayer surface, a significant alteration in the band structure is observed. As depicted in Figure 3, four primary impurity levels originating from the As atoms emerge between the valence band maximum and the conduction band minimum. The introduction of these impurity levels substantially narrows the original band gap, reducing the new band gap of MoS_2_ post-As adsorption to 0.242 eV. This represents an approximate 84.7% decrease compared to the baseline state, suggesting a marked enhancement in the electrical conductivity of the MoS_2_-As adsorption system, thereby improving its efficiency as a heavy metal sensor.

On the other hand, the adsorption of Cd or Pb on the MoS_2_ monolayer introduces a new impurity level below the Fermi energy, resulting in band gaps of the MoS_2_-Cd and MoS_2_-Pb adsorption systems being reduced to 1.14 eV and 0.326 eV, respectively. Compared to the baseline state, the band gaps have been reduced by 28.1% and 79.4%, respectively. While adsorption in both cases promotes an increase in conductivity, the extent is not as significant as with As adsorption. In contrast, Hg atom adsorption on the MoS_2_ monolayer does not induce noticeable impurity level formation, implying that post-Hg adsorption, the band gap of the MoS_2_ monolayer decreases by only 3.2%. This suggests that Hg adsorption has a minimal impact on the change in conductivity of the MoS_2_ monolayer, and compared to the adsorption of other elements, it does not significantly alter its conductive response characteristics as a sensor.

**Figure 4:**
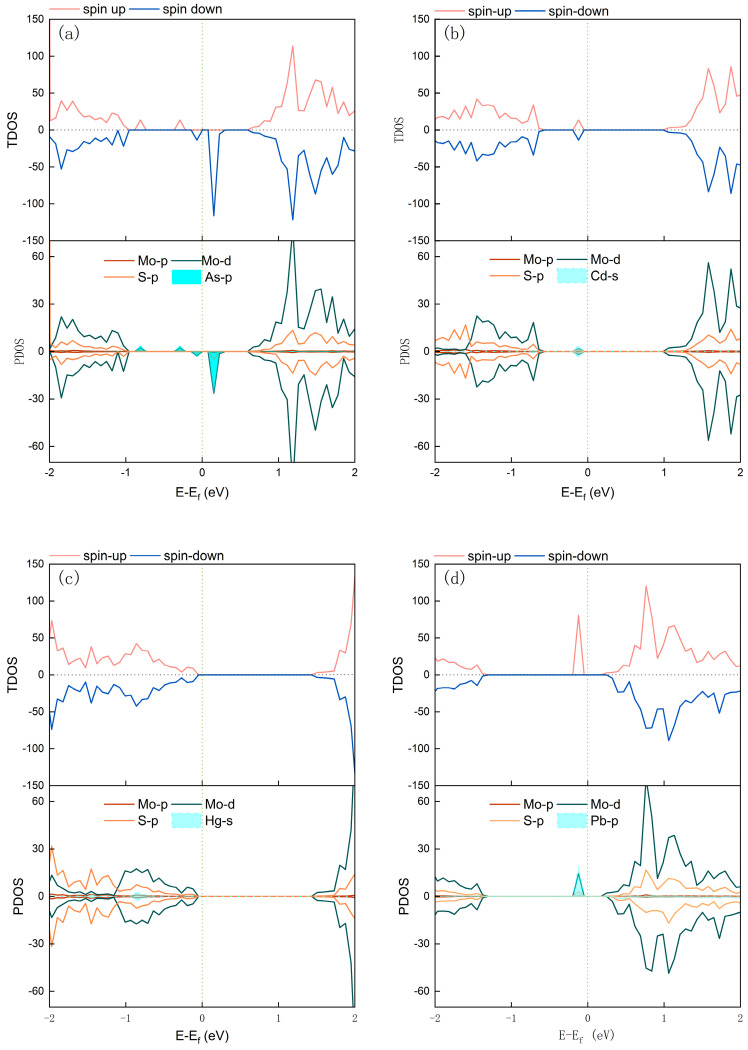
Total and partial DOS of MoS_2_ monolayer: (**a**) As, (**b**) Cd, (**c**) Hg, and (**d**) Pb adsorption (Fermi level is set as 0 eV).

To further elucidate the adsorption characteristics of MoS_2_ towards heavy metals, the impact of heavy metal adsorption on the electronic properties of MoS_2_ was investigated by analyzing the total density of states (TDOS) and partial density of states (PDOS) as shown in Figure 4.

After As adsorption on MoS_2_, four new hybridized energy levels were generated near the Fermi level. These levels correspond to spin-up peaks at −0.809 eV and −0.292 eV, a spin-down peak at −0.070 eV, and a sharp, narrow peak just above the Fermi level at 0.152 eV. The p-orbitals of As play a dominant role in the formation of these hybridized levels, simultaneously inducing peak states in the density of states for Mo’s d-orbitals and S’s p-orbitals at corresponding energy positions. These results demonstrate strong orbital hybridization between As and the surface atoms of MoS_2_, and due to the asymmetry of the DOS curves for spin-up and spin-down, it is evident that As adsorption imparts magnetism to the MoS_2_ system, with a calculated magnetic moment of 1 µB.

For Cd adsorption on MoS_2_, a DOS peak is observed at −0.121 eV, resulting from the hybridization of Cd’s s-orbital electrons with Mo’s d-orbital electrons and S’s p-orbital electrons. However, Cd adsorption does not lead to significant spin polarization, as the DOS curves remain symmetrical for spin-up and spin-down, indicating that the Cd adsorption system is non-magnetic.

After Pb adsorption on MoS_2_, a DOS peak is observed at −0.121 eV, primarily attributed to the cooperative effect of Pb’s p-orbital electrons with Mo’s d-orbital electrons and S’s p-orbital electrons. Similar to As adsorption, the system post-Pb adsorption exhibits asymmetry in the DOS curves for spin-up and spin-down, resulting in a magnetic moment of 2 µB, confirming the magnetic nature of the Pb adsorption system.

As a result, As, Cd, and Pb all exhibit a certain degree of orbital hybridization effects during their interaction with MoS_2_. The adsorption of As and Pb leads to a transition of the MoS_2_ system to a magnetic state, suggesting that MoS_2_ holds promise as a magnetic sensing material for As and Pb detection, while the detection of Cd and Hg does not depend on a magnetic response mechanism.

**Figure 5:**
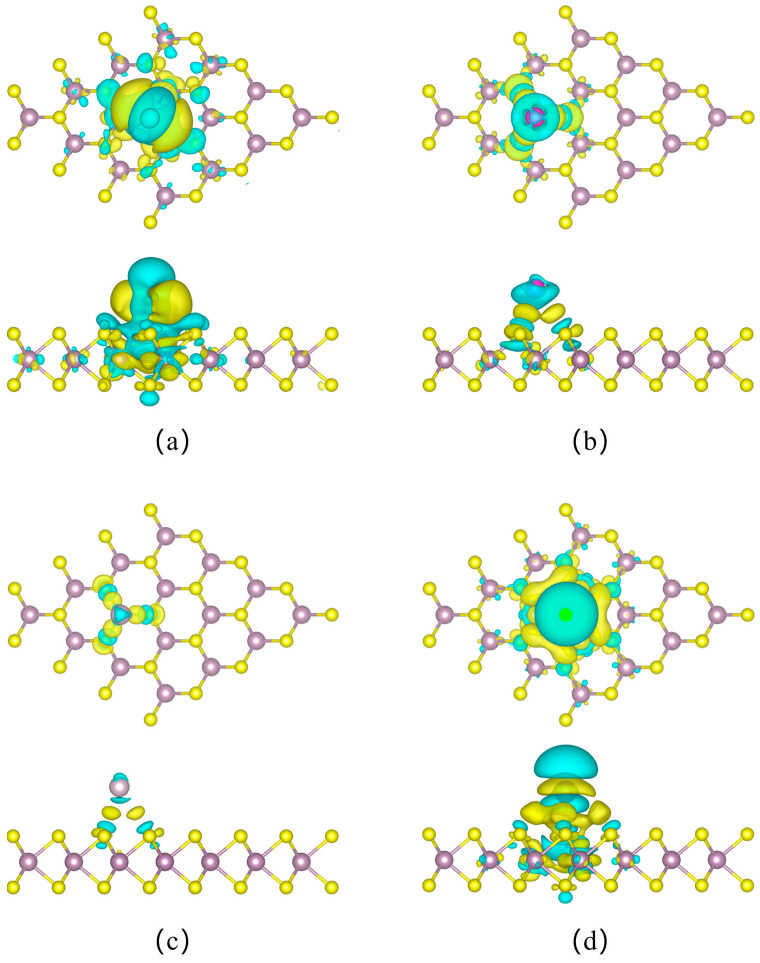
Charge density differential results for (**a**) As, (**b**) Cd, (**c**) Hg, and (**d**) Pb adsorbed on monolayer MoS_2_. The isosurface value is taken as 0.0003 e/Å^3^.

To gain deeper insight into the bonding characteristics between the adsorbed metal atoms and the MoS_2_ monolayer surface, we analyzed the differential charge density. This quantity is calculated using Equation (3):

(3) $$\begin{array}{c} ∆\rho =\rho_{total}-\rho_{{MoS}_{2}}-\rho_{metal} \end{array}$$

where ∆ρ represents the charge density difference, $\rho_{total}$ is the charge density of the combined adsorption system, $\rho_{{MoS}_{2}}$ is the charge density of the pristine MoS_2_ monolayer, and $\rho_{metal}$ is the charge density of the isolated adsorbate atom. Figure 5 illustrates the differential charge density, with regions of electron accumulation depicted in yellow and areas of electron depletion shown in blue.

Through the study of As and Pb adsorption on the MoS_2_ surface, significant regions of charge exchange were identified within these adsorption systems, as further confirmed by Bader charge analysis depicted in Table 1. Notably, As and Pb exhibited substantial charge transfers to the MoS_2_, with values of −0.243 e and −0.410 e, respectively. In contrast, Cd exhibits a relatively weaker charge exchange with the MoS_2_ surface, with a value of −0.115 e. The charge transfer between Hg and MoS_2_ is even more negligible, amounting to only −0.023 e.

### 3.3. Recovery Time

**Figure 6:**
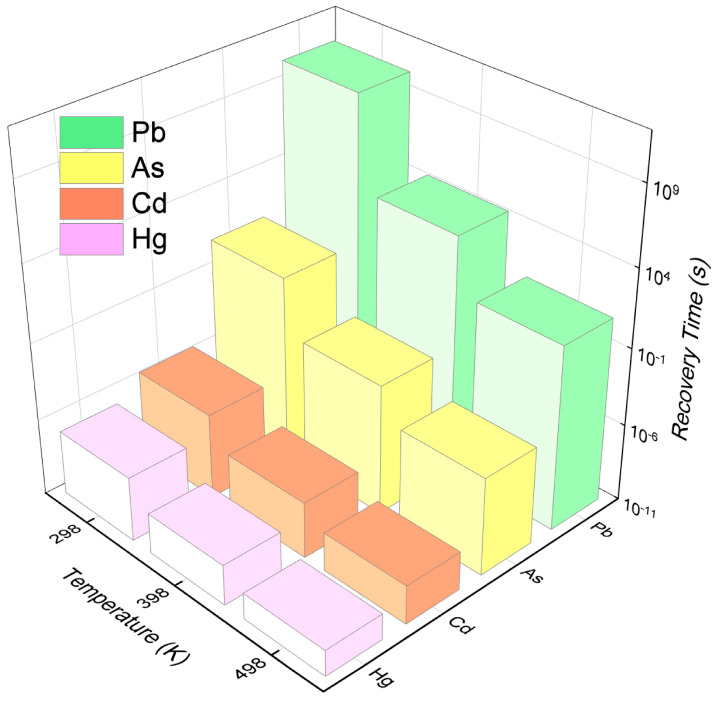
The recovery time (τ) for As, Cd, Hg, and Pb adsorbed on MoS_2_ monolayers at different temperatures (T).

The recovery time is a crucial property for sensor performance as it determines the speed and repeatability of the sensor’s response. The recovery time (τ) represents the time needed for the sensor surface to restore its initial adsorption capacity after becoming saturated with adsorbates. The recovery time, denoted by (τ), is deduced from Equation (4), which is an extrapolation from classical transition state theory references:

(4) $$\begin{array}{c} \tau =\exp\left(\frac{-E_{ad}}{k_{B}T}\right)/\omega \end{array}$$

where ω signifies the attempt frequency, postulated to be 10^13^ s^−1^ [32]. E_ad_ represents the adsorption energy, k_B_ is the Boltzmann constant, and T denotes the temperature. After adsorption of various heavy metals on MoS_2_, the calculated recovery times within the 298–498 K range are presented in Table S1. As delineated in Figure 6, the recovery times for heavy metals on the MoS_2_ monolayer are inversely proportional to the temperature increment. At 298 K, the desorption times for As, cadmium (Cd), and Hg from the MoS_2_ monolayer surface are 9.67 s, 3.54 × 10^−6^ s, and 1.56 × 10^−7^ s, respectively, indicating a swift recovery performance at ambient conditions. For Pb, the recovery time on a MoS_2_ monolayer reduces to 2.21 × 10^10^ s at 298 K, and further to 9.32 s at an elevated temperature of 498 K. This observation suggests that the MoS_2_ monolayer, when employed as a Pb sensor, exhibits reusability at the heightened temperature of 498 K. Consequently, the MoS_2_ monolayer emerges as a promising candidate for sensing As, Cd, Hg, and Pb, attributable to its expedited recovery dynamics.

### 3.4. Sensitivity

**Figure 7:**
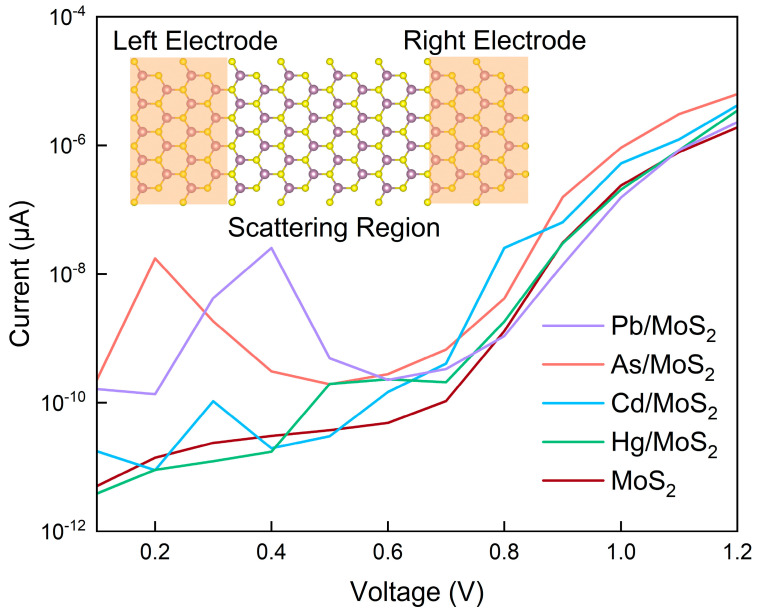
The current-voltage characteristics of MoS_2_ monolayers with As, Cd, Hg, and Pb adsorption.

The electrical transport properties of the MoS_2_ monolayer upon heavy metal adsorption were investigated by constructing a double-probe model, comprising two electrodes with an intervening scattering region, as depicted in Figure 7. A bias voltage was applied across the electrodes. This quantum transport problem was solved using the NEGF-DFT method under open boundary conditions and in the presence of external electric fields.

Figure 7 also delineates the current-voltage (I-V) characteristics of MoS_2_ monolayers, both in pristine form and following the adsorption of heavy metals. At a bias voltage of 0.2 V, the MoS_2_ monolayer with adsorbed As demonstrated a significantly enhanced current, reaching 1.75 × 10^−8^ µA, which is markedly higher than the basal current of intrinsic MoS_2_ at 1.39 × 10^−11^ µA. Comparatively, the currents observed for MoS_2_ monolayers with adsorbed Cd, Hg, and Pb were 8.92 × 10^−12^ µA, 9.01 × 10^−12^ µA, and 1.35 × 10^−10^ µA, respectively, indicating a pronounced selectivity for arsenic.

For Pb adsorption, the current at low bias voltages (≤0.6 V) was markedly higher than that of intrinsic MoS_2_, while at higher bias voltages (≥0.8 V), the disparity was considerably reduced. Notably, at a bias voltage of 0.4 V, the MoS_2_ monolayer with adsorbed arsenic (As) manifested a current of 2.54 × 10^−8^ µA, which is considerably higher than the currents measured for MoS_2_ monolayers with adsorbed As (3.07 × 10^−10^ µA), Cd (1.95 × 10^−11^ µA), Hg (1.72 × 10^−11^ µA), and the intrinsic MoS_2_ (3.04 × 10^−11^ µA).

The MoS_2_ monolayer with Hg adsorption showed a relatively minor difference in current from intrinsic MoS_2_, except in the 0.5–0.6 V range. The current for Cd-adsorbed MoS_2_ was generally lower than that for As, but higher than for Hg.

The sensitivity (S) of MoS_2_ monolayers was also evaluated using Equation (5):

(5) $$\begin{array}{c} S=\left|I-I_{0}\right|/I_{0} \end{array}$$

where I represents the current measured across the MoS_2_ monolayer upon heavy metal adsorption, and I_0_ denotes the intrinsic current in the absence of heavy metal contaminants.

**Figure 8:**
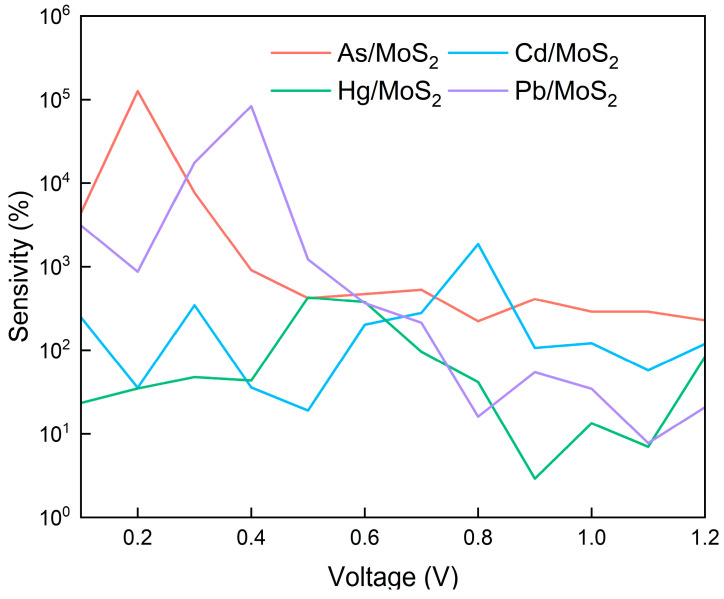
The sensitivity for As, Cd, Hg, and Pb adsorbed on MoS_2_ monolayers.

As illustrated in Figure 8, the sensitivity of the MoS_2_-based monolayer sensors can be quantitatively estimated. Upon adsorption of As, the MoS_2_ sensor exhibits a remarkable sensitivity, reaching 126,452.28% at a bias voltage of 0.2 V. This sensitivity is significantly higher compared to other heavy metals, indicating a pronounced selectivity for As. Moreover, the MoS_2_ sensor maintains a sensitivity above 200% across the entire voltage range below 1.2 V after As adsorption, demonstrating exceptional sensitivity and superior sensing performance.

In the case of Pb, the MoS_2_ sensor achieves a sensitivity of 83,438.90% at 0.4 V, which is considerably higher than that for other heavy metals, suggesting good selectivity at this voltage. Within a voltage range of ≤0.7 V, the sensitivity for Pb remains above 200%.

For Cd, the peak sensitivity of 1862.67% is reached at 0.8 V, with the sensitivity exceeding 200% at bias voltages of 0.1 V, 0.3 V, and 0.6–0.8 V. Conversely, the sensitivity for Hg is relatively lower, peaking at 427.71% at 0.5 V and surpassing 200% within the range of 0.5–0.6 V.

The limit of detection (LOD) signifies the lowest analyte concentration a sensor can reliably detect. Determined via signal-to-noise ratio (SNR), LOD compares the analyte signal to a blank signal to establish the minimum detectable concentration. For MoS_2_ sensors, current serves as the output signal. Baseline current (I_0_) represents the MoS_2_ current without heavy metal adsorption, while current (I) reflects the post-adsorption state.

(6) $$\begin{array}{c} S=\left|I-I_{0}\right|/I_{0}=\left|I//I_{0}-1\right|=\left|\mathrm{SNR}-1\right| \end{array}$$

Conventionally, LOD corresponds to an SNR of 3:1, which also translates to a sensitivity of 200%.

To quantify heavy metal adsorption on MoS_2_ monolayers, concentration is expressed as the atomic count per area. The calculations above employed a 25.54 Å × 22.12 Å scattering region (564.94 Å^2^). A single heavy metal atom within this area (concentration: 1/564.94 Å^2^) yields MoS_2_ sensitivities surpassing the LOD for As (126,452.28%), Cd (1862.67%), Hg (427.71%), and Pb (83,438.90%).

Doubling the scattering region while maintaining a single metal atom reduces concentration to 1/1129.88 Å^2^. Under these conditions, the sensitivities of MoS_2_ towards As, Cd, Hg, and Pb are 132.33%, 215.42%, 122.38%, and 828.19%, respectively. Here, MoS_2_ sensitivity significantly exceeds the LOD for Pb, marginally surpasses it for Cd, but falls below for As and Hg. Consequently, MoS_2_ detects Pb at concentrations below 1/1129.88 Å^2^, Cd near 1/1129.88 Å^2^, and As and Hg between 1/1129.88 Å^2^ and 1/564.94 Å^2^.

These findings underscore the high sensitivity and selectivity of MoS_2_-based monolayer sensors towards heavy metals, positioning them as promising candidates for future environmental monitoring applications.

**Figure 9:**
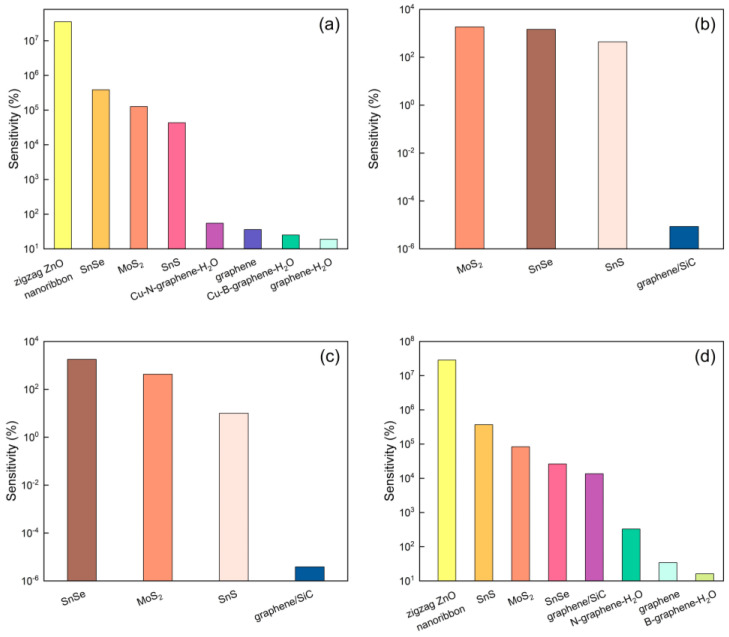
The comparison of sensitivity for (**a**) As, (**b**) Cd, (**c**) Hg, and (**d**) Pb sensing materials.

A comparative investigation of different two-dimensional nanomaterials’ sensitivity to heavy metal detection [7,8,9,10,11,22,33,34,35,36,37,38,39,40] has been conducted in this study. The data are clearly listed in Table S2 and are presented in graphical form in Figure 9. As depicted in Figure 9a, the MoS_2_ monolayer exhibits a sensitivity of 126,452.28% to As. Although this is slightly less than that of zigzag ZnO nanoribbons (3.52 × 10^7^%) [33] and SnSe (384,467%) [34], it is significantly superior to SnS (43,286%) [35], Cu-N-doped graphene-H_2_O (55%) [36], graphene (36.06%) [37], Cu-B-doped graphene-H_2_O (25%) [36], and graphene-H_2_O (19%) [36]. Additionally, MoS_2_ demonstrates a shorter recovery time under ambient conditions, facilitating rapid and continuous detection. In contrast, zigzag ZnO nanoribbons are lacking in adsorption energy data, which hinders the assessment of their reusability at room temperature.

As shown in Figure 9b, upon further investigation of Cd sensing, MoS_2_ demonstrates the highest sensitivity reported to date, reaching 1862.67%, exceeding SnSe (1462%) [34] and SnS (442%) [35], and far outperforming the graphene/SiC composite material (8.5702 × 10^−8^%) [9]. For Hg detection, although the sensitivity of MoS_2_ is 427.71%, lower than SnSe (1791%) [34], it is still significantly higher than SnS (10%) [35] and graphene/SiC (3.868 × 10^−8^%) [9], as Figure 9c illustrates.

In the context of Pb sensing depicted by Figure 9d, the sensitivity of MoS_2_ reaches 83,438.90%, which is second only to zigzag ZnO nanoribbons (2.86 × 10^7^%) [33] and SnS (368,860%) [35], but far exceeds SnSe (26,160%) [34], graphene/SiC (13,600%) [9], N-doped graphene-H_2_O (329.04%), and B-doped graphene-H_2_O (16.12%) [38]. Notably, the recovery time of MoS_2_ at 498 K is only 9.32 s, compared to SnS, which requires 76.44 s at 598 K, and SnSe, which takes 38.3 s at 498 K to recover fully. This underscores the rapid response capability of MoS_2_.

A critical advantage of MoS_2_ monolayer sensors over other 2D material-based heavy metal sensors lies in their ability to selectively detect specific heavy metals in complex samples. Unlike other 2D materials, such as tin disulfide (SnS) and tin selenide (SnSe), where multiple heavy metals exhibit similar response peaks, MoS_2_ displays unique response peaks for each heavy metal.

As illustrated in Table S2, SnS exhibits response peaks for Cd, Hg, and Pb at the same voltage (0.5 V), leading to potential interference issues. In samples containing multiple heavy metals, the signal of more sensitive metals like Pb, Cd, and Hg can be masked by the stronger response of Pb. Similarly, SnSe exhibits response peaks for As, Cd, Hg, and Pb at distinct bias voltages (0.2 V, 0.1 V, 0.1 V, and 0.2 V, respectively). However, in mixed heavy metal samples, Cd and Hg, as well as As and Pb, can still interfere with each other.

In contrast, MoS_2_ demonstrates remarkable selectivity, with distinct response peaks for different heavy metals at varying bias voltages. For instance, As, Cd, Hg, and Pb exhibit response peaks at 0.2 V, 0.8 V, 0.5 V, and 0.4 V, respectively. This unique characteristic allows for the selective detection of specific heavy metals by adjusting the working voltage applied across the MoS_2_ monolayer.

## 4. Conclusions

The sensing performance of the MoS_2_ monolayer on heavy metals (As, Cd, Hg, and Pb) is investigated using DFT and NEGF simulations. As and Pb adsorption onto MoS_2_ monolayers causes significant surface structural modifications and intensive electrical interactions. These interactions introduce impurity energy levels within the adsorption system, leading to significant band gap alterations. Conversely, the MoS_2_ surface is less affected by Cd and Hg, as evidenced by a weaker electronic exchange. The sensitivities of MoS_2_ monolayers to As, Cd, Hg, and Pb are 126,452.28%, 1862.67%, 427.71%, and 83,438.90%, respectively. For As or Pb, the MoS_2_ monolayer sensors exhibit response peaks at different bias voltages that are significantly higher than those for other heavy metals, demonstrating a high degree of selectivity. When As or Pb are adsorbed on MoS_2_ monolayers, they acquire a non-zero magnetic moment of 1 µB or 2 µB, respectively, indicating that they may find use as magnetic sensors. The MoS_2_ monolayer’s moderate adsorption energy facilitates rapid sensor recovery for As, Hg, and Cd at room temperature. Furthermore, the recovery time for Pb can be remarkably reduced to just 9.32 s at 498 K, highlighting the MoS_2_ monolayer’s efficacy as a reusable sensor. The results of this study show that MoS_2_ monolayer has the potential to be a high sensitivity, selectivity, and reusability heavy metal sensing material that can be used to make heavy metal detectors that operate in real-time.

## Figures and Tables

**Table 1:** Adsorption energy, adsorption distance, charge transfer, and energy gap.

Heavy Metal	Adsorption Energy Ead (eV)	Adsorption Distance D (Å) ^1^	Charge Transfer △Q (e)	Style	Energy Gap Eg (eV)
As	−0.827	2.175	−0.243	Donor	0.242
Cd	−0.446	3.358	−0.115	Donor	1.140
Hg	−0.366	3.526	−0.023	Donor	1.534
Pb	−1.380	2.625	−0.410	Donor	0.326

^1^ The adsorption distance is defined as the closest contact distance between the adsorbed atom and the nearest atom on the MoS_2_ surface.

## Data Availability

The original contributions presented in the study are included in the article, further inquiries can be directed to the corresponding author.

## Supplementary Materials

The following supporting information can be downloaded at: https://www.mdpi.com/article/10.3390/mi15080978/s1, Table S1: Temperature-dependent recovery time of MoS_2_ for heavy metal adsorption; Table S2: Comparison between this work and previous reports.
