# Supplementary material for: First-Principles Insights into Highly Sensitive and Reusable MoS2 Monolayers for Heavy Metal Detection
Source: Micromachines (Basel). 2024 Jul 30;15(8):978. doi: 10.3390/mi15080978 (PMC11355996; doi:10.3390/mi15080978)
Supplement: Supplementary file 1 [file micromachines-15-00978-s001.zip › micromachines-3096957-supplementary.pdf]

First-Principles Insights into Highly Sensitive and Reusable MoS_2 Monolayers for Heavy Metal Detection

Jiayin Wu [1,2], Zongbao Li [3,4,*], Tongle Liang [5], Qiuyan Mo [6], Jingting Wei [1], Bin Li [1] and Xiaobo Xing [2,*]

1 Department of Engineering Technology, Guangdong Open University, Guangzhou 510091, China; wujiayin@m.scnu.edu.cn (J.W.); bli@gdrtvu.edu.cn (B.L.); jtwei@gdrtvu.edu.cn (J.W.)
2 Centre for Optical and Electromagnetic Research, South China Academy of Advanced Optoelectronics, South China Normal University, Guangzhou 510006, China
3 Ministry of Education Key Laboratory of Textile Fiber Products, School of Materials Science and Engineering, Wuhan Textile University, Wuhan 430220, China
4 School of Materials and Chemistry Engineering, Tongren University, Tongren 554300, China
5 School of Artificial Intelligence, Guangdong Vocational College of Post and Telecom, Guangzhou 510630, China; liangtongle@gupt.edu.cn
6 Big Data Engineering College, Kaili University, Kaili 556011, China; 2014020983@kluniv.edu.cn
* Correspondence: zongbaoli1982@163.com (Z.L.); xingxiaobo@scnu.edu.cn (X.X.)

Table S1: Temperature-dependent recovery time of MoS_2 for heavy metal adsorption

Heavy metal	Temperature	Recovery time
As	298K	9.67 s
As	398K	2.96×10^{-3} s
As	498K	2.34×10^{-5} s
Cd	298K	3.54×10^{-6} s
Cd	398K	4.49×10^{-8} s
Cd	498K	3.29×10^{-9} s
Hg	298K	1.56×10^{-7} s
Hg	398K	4.33×10^{-9} s
Hg	498K	5.07×10^{-10} s
Pb	298K	2.21×10^{10} s
Pb	398K	3.02×10^{4} s
Pb	498K	9.32 s

Table S2: Comparison between this work with previous reports

Sensing material	Heavy metal	Adsorption energy E_{ad}(eV)	Recovery time, temperature	Sensitivity, bias voltage [a]	Ref
MoS_2	As	-0.827	9.67 s, 298K	126452.28%,0.2V	This work
SnS	As	-1.599	2.99 s, 598K	43286%,0.3V	[1]
SnSe	As	-0.663	0.016 s, 298K	384467%,0.2V	[2]
graphene	As	-1.39		36.06%, 0.6V	[3]
graphene -H_2O	As	-0.97			[3]
boron functionalized graphene	As	-2.63			[4]
boron functionalized graphene-H_2O	As	-1.91			[4]
graphene-H_2O	As	-1.73		19%, 0.2V	[5]
Cu-B-graphene-H_2O	As	-3.43		25%, 0.2V	[5]
Cu-N-graphene-H_2O	As	-3.93		55%, 0.2V	[5]
zigzag ZnO nanoribbons	As			3.52×10^{7}, 0.3V	[6]
MoS_2	Cd	-0.446	3.54×10^{-6} s, 298K	1862.67%,0.8V	This work
SnS	Cd	-0.384	31.2 μs, 298K	442%,0.5V	[1]
SnSe	Cd	-0.427	1.67×10^{-6} s, 298K	1462%,0.1V	[2]
graphene	Cd	-0.23			[3]
graphene -H_2O	Cd	-0.31			[3]
Epitaxial graphene	Cd	0.217			[7]
Epitaxial graphene-H_2O	Cd	0.293			[7]
zinc-oxide nanotube	Cd	−1.30			[8]
zinc-oxide graphene-like structure	Cd	−0.24			[8]
single vacancy graphene	Cd	−0.409			[9]
graphene/SiC	Cd			8.5702×10^{-8}, 1V	[10]
MoS_2	Hg	-0.366	1.56×10^{-7} s, 298K	427.71%,0.5V	This work
SnS	Hg	-0.473	9.986 μs, 298K	10%,0.5V	[1]
SnSe	Hg	-0.278	5.03×10^{-9} s, 298K	1791%, 0.1V	[2]
graphene	Hg	-0.26			[3]
graphene -H_2O	Hg	-0.32			[3]
Epitaxial graphene	Hg	0.246			[7]
Epitaxial graphene-H_2O	Hg	0.384			[7]
graphene/SiC	Hg			3.868×10^{-8}, 1V	[10]
Ti_2C	Hg	-0.735	28.8 s,400K		[11]
1T-MoS_2	Hg	-1.091			[12]
Pd_2-pyridine-like N -graphene	Hg	-1.02			[13]
MoS_2	Pb	-1.380	9.32 s, 498K	83438.90%,0.4V	This work
SnS	Pb	-1.766	76.44 s, 598K	368860%, 0.5V	[1]
SnSe	Pb	-1.441	38.3 s, 498K	26160%,0.2V	[2]

graphene	Pb	-0.91		34.42%, 0.6V	[3]
graphene -H_2O	Pb	-0.97			[3]
zigzag ZnO nanoribbons	Pb			2.86×10^{7}, 0.2V	[6]
Epitaxial graphene	Pb	0.641			[7]
Epitaxial graphene-H_2O	Pb	1.57			[7]
graphene/SiC	Pb			13600%, 1V	[10]
B dope- graphene	Pb	-2.40	1.4×10^{14} μs,600K		[14]
N dope- graphene	Pb	-1.01	302 μs,600K		[14]
B dope -graphene-H_2O	Pb	-1.73	3.31×10^{8} μs,600K	16.12%,0.15V	[14]
N dope -graphene-H_2O	Pb	-0.95	8.63 μs,600K	329.04%,0.15V	[14]

References

1. Wu, J.; Li, X.; Liao, H.; Xue, S.; Huang, L.; Xing, X.; Luo, A. SnS Monolayers Based Heavy Metal Sensors: DFT and NEGF Analysis. *Results in Physics* **2023**, *51*, 106704, doi:10.1016/j.rinp.2023.106704.
2. Wu, J.; Li, X.; Huang, L.; Liang, T.; Xing, X.; Luo, A. SnSe Monolayer-Based Heavy Metal Sensors with High Sensitivity, Selectivity, and Reusability: Insights from First Principle Calculation. *Results in Physics* **2023**, 106973, doi:10.1016/j.rinp.2023.106973.
3. Srivastava, M.; Srivastava, A.; Pandey, S.K. Suitability of Graphene Monolayer as Sensor for Carcinogenic Heavy Metals in Water: A DFT Investigation. *Applied Surface Science* **2020**, *517*, 146021, doi:10.1016/j.apsusc.2020.146021.
4. Srivastava, M.; Srivastava, A. First Principle Investigation of Boron Functionalized Graphene to Detect the Presence of "Arsenic" in Water. *Materials Today: Proceedings* **2022**, *48*, 661–665, doi:10.1016/j.matpr.2021.07.147.
5. Srivastava, M.; Srivastava, A. Cu Decorated Functionalized Graphene for Arsenic Sensing in Water: A First Principles Analysis. *Applied Surface Science* **2021**, *560*, 149700, doi:10.1016/j.apsusc.2021.149700.
6. Sankush Krishna, M.; Singh, S.; Mohammed, M.K.A. Carcinogenic Heavy Metals Detection Based on ZnO Nanoribbons. *IEEE Sensors J.* **2022**, 1–1, doi:10.1109/JSEN.2022.3192928.
7. Shtepliuk, I.; Yakimova, R. Interaction of Epitaxial Graphene with Heavy Metals: Towards Novel Sensing Platform. *Nanotechnology* **2019**, *30*, 294002, doi:10.1088/1361-6528/ab1546.
8. Hamed Mashhadzadeh, A.; Fathalian, M.; Ghorbanzadeh Ahangari, M.; Shahavi, M.H. DFT Study of Ni, Cu, Cd and Ag Heavy Metal Atom Adsorption onto the Surface of the Zinc-Oxide Nanotube and Zinc-Oxide Graphene-like Structure. *Materials Chemistry and Physics* **2018**, *220*, 366–373, doi:10.1016/j.matchemphys.2018.09.016.
9. Liao, J.; Yang, L.; Wang, C.Z.; Lin, S. Tuning Cd Adsorption Behaviours on Graphene by Introducing Defects: A First-Principles Study. *Materials Technology* **2017**, *32*, 840–844, doi:10.1080/10667857.2017.1304608.

10. Shtepliuk, I.; Eriksson, J.; Khranovskyy, V.; Iakimov, T.; Lloyd Spetz, A.; Yakimova, R. Monolayer Graphene/SiC Schottky Barrier Diodes with Improved Barrier Height Uniformity as a Sensing Platform for the Detection of Heavy Metals. *Beilstein J. Nanotechnol.* **2016**, *7*, 1800–1814, doi:10.3762/bjnano.7.173.
11. Yang, T.; Jiang, X.; Yi, W.; Cheng, X.; Cheng, T. Enhanced Fast Response to Hg0 by Adsorption-Induced Electronic Structure Evolution of Ti2C Nanosheet. *Applied Surface Science* **2021**, *544*, 148925, doi:10.1016/j.apsusc.2021.148925.
12. Mu, X.; Gao, X.; Zhao, H.; George, M.; Wu, T. Density Functional Theory Study of the Adsorption of Elemental Mercury on a 1T-MoS2 Monolayer. *J. Zhejiang Univ. Sci. A* **2018**, *19*, 60–67, doi:10.1631/jzus.A1700079.
13. Zhao, C.; Wu, H. Density Functional Investigation of Mercury and Arsenic Adsorption on Nitrogen Doped Graphene Decorated with Palladium Clusters: A Promising Heavy Metal Sensing Material in Farmland. *Applied Surface Science* **2017**, *399*, 55–66, doi:10.1016/j.apsusc.2016.12.084.
14. Srivastava, M.; Srivastava, A. DFT Analysis of Nitrogen and Boron Doped Graphene Sheet as Lead Detector. *Materials Science and Engineering: B* **2021**, *269*, 115165, doi:10.1016/j.mseb.2021.115165.
